# CAN CHATGPT PERFORM GERIATRIC KNOWLEDGE TESTS AS WELL AS MEDICAL STUDENTS, RESIDENTS, AND GERIATRICS FELLOWS?

**DOI:** 10.1093/geroni/igae098.2989

**Published:** 2024-12-31

**Authors:** Huai Cheng

**Affiliations:** Minneapolis VA Hospital, Playmouth, Minnesota, United States

## Abstract

ChatGP was released to the public in November of 2022 and immediately emerged as a quick information resource and application to medical education. ChatGPT passed USMLE step 1, 2 and 3. This study was to examine whether ChatGPT can perform well on 18 published validated geriatric knowledge questions compared to medical students, residents, and geriatrics medicine fellows. These 18 geriatric knowledge questions with 8 True or False questions and 10 clinical vignettes were developed to assess geriatric knowledge for medical students, residents, and geriatrics medicine fellows. In this study, ChatGPT was treated as a human or study participant and prompted to answer these 18 questions four times from April to November of 2023. Outputs from ChatGPT were collected for analysis. The mean scores of 18 questions were compared between ChatGPT and the trainees including medical students, residents, and geriatrics medicine fellows. It was found that the mean score (14.25, n=4 prompts) from ChatGPT was higher than the scores from medical students and similar to residents, however lower than geriatrics medicine fellows. Additionally, half of output from ChatGPT gave good rational for their response to the questions. This indicated ChatGPT performed reasonably well on 18 validated geriatric knowledge questions. ChatGPT could be used as a supplemental resource in geriatrics for trainees at their early stage of training and perhaps for lay persons as well.

